# Sex‐specific associations between regular exercise habits and serum myokine levels in adults with obesity

**DOI:** 10.14814/phy2.70930

**Published:** 2026-05-27

**Authors:** Satoshi Kurose, Katsuko Onishi, Takumi Miyauchi, Kazuhisa Takahashi, Yutaka Kimura

**Affiliations:** ^1^ Faculty of Sport and Health Sciences Osaka Sangyo University Daito Osaka Japan; ^2^ Health Science Center Kansai Medical University Hirakata Osaka Japan; ^3^ Department of Medicine II Kansai Medical University Hirakata Osaka Japan

**Keywords:** exercise habits, myokine, obesity, sex difference

## Abstract

Adults with obesity have increased fat mass and greater absolute skeletal muscle mass, which may confound interpretation of exercise‐induced myokine responses. This study examined sex‐specific differences in myokine dynamics based on exercise habits in adults with obesity matched for body composition. This retrospective, sex‐stratified, matched cross‐sectional study included adults with obesity (body mass index ≥30 kg/m^2^) attending an obesity clinic. Eighty participants (20 males and 20 females in each exercise habit group) were matched for age, body weight, body mass index, body fat mass and lean body mass. Regular exercise habits were defined as ≥30 min at least twice weekly. Serum concentrations of myostatin (MST), follistatin, irisin, brain‐derived neurotrophic factor, follistatin‐like protein 1 (FSTL1), and secreted proteins acidic and rich in cysteine were measured. A significant interaction between sex and exercise habits was observed for serum FSTL1 levels (*p* = 0.023), whereas MST showed significant main effects of both sex and exercise habits. Multiple regression analysis revealed that daily physical activity in females was an independent determinant of MST (β = −0.400, *p* = 0.011) and FSTL1 (β = −0.417, *p* = 0.007). These findings suggest that daily physical activity is an independent determinant of circulating MST and FSTL1 levels in females with obesity, highlighting sex‐specific differences in myokine regulation.

## 1 INTRODUCTION

Obesity is a fundamental condition underlying many lifestyle‐related diseases and contributes to tumor cell proliferation and atherosclerosis through adipokine dysregulation, insulin resistance, and chronic inflammation induced by adipocyte hypertrophy (Hruby & Hu, 2015; Yatsuya et al., 2010). The prevalence of obesity is increasing worldwide (NCD Risk Factor Collaboration (NCD‐RisC), 2017), highlighting the need for early therapeutic intervention to prevent disease progression and severe complications. Although previous studies have shown that obesity‐related parameters improve significantly with a weight reduction of ≥3% (Kurose et al., 2014; Muramoto et al., 2014), weight loss interventions are often associated with reductions in skeletal muscle mass, particularly when not combined with resistance training (Tanaka et al., 2019). Notably, adults with obesity who experience loss of skeletal muscle mass during weight reduction have been reported to exhibit cardiovascular risk profiles comparable to those of adults with obesity who regain weight, underscoring the importance of preserving skeletal muscle mass during weight loss (Cai et al., 2024).

In recent years, skeletal muscle has attracted increasing attention not only as a component of the musculoskeletal system but also as an endocrine organ that secretes myokines (Pedersen, 2013). To date, >650 myokines have been proposed, and novel myokines have been identified (Severinsen & Pedersen, 2020). Although only several dozen myokines can be reliably measured in human serum, they are known to exert autocrine and paracrine effects on skeletal muscle and endocrine effects on distant organs throughout the body (Severinsen & Pedersen, 2020). Among the myokines involved in the regulation of body composition, myostatin (MST), which suppresses muscle growth; follistatin (FST), which inhibits MST and promotes muscle hypertrophy; and irisin, which induces browning of white adipose tissue, are well established (Boström et al., 2012; Mather et al., 1997; McPherron et al., 1997). Furthermore, skeletal muscle–derived myokines include brain‐derived neurotrophic factor (BDNF), which regulates neuroprotection and muscle metabolism; follistatin‐like protein 1 (FSTL1), which promotes myocardial protection and angiogenesis; and secreted protein acidic and rich in cysteine (SPARC), which exhibits antitumor effects (Aoi et al., 2013; Miyabe et al., 2014; Shimano et al., 2011; Wrann et al., 2013). These myokines are characterized by either the constitutive or dynamic regulation of their secretion in response to exercise. Exercise is a key regulator of myokine secretion, with both acute and chronic exercise shown to modulate circulating myokine levels and influence metabolic health (Domin et al., 2021). Our previous study demonstrated that, in a weight‐loss program for adults with obesity, the secretion patterns of MST and FST varied depending on the rate of weight loss (Kurose et al., 2024). Furthermore, exercise tolerance and sedentary behavior were identified as factors associated with these changes. However, the relationship between regular exercise habits and myokine profiles, particularly in adults with obesity, remains incompletely understood.

Although myokines are cytokines and peptides released from skeletal muscle, some of these molecules can also be expressed and secreted by other tissues (Deng et al., 2017; Flanagan et al., 2009; Kos & Wilding, 2010; Xu et al., 2020). Notably, adults with obesity typically exhibit both increased fat mass and skeletal muscle mass, suggesting that muscle–adipose tissue crosstalk plays a role in myokine dynamics; however, the underlying mechanisms remain unclear (Kurose et al., 2021). To accurately evaluate the effects of exercise on myokines, it is essential to conduct observations in adults with obesity who have comparable body composition. Many myokines are released into the circulation in response to skeletal muscle contraction and exert endocrine effects on multiple target organs, including adipose tissue, liver, endocrine system, and vasculature, thereby contributing to systemic homeostasis (So et al., 2014). Consequently, myokines have attracted considerable attention as key molecular mediators in exercise‐induced disease prevention and treatment. Although numerous clinical studies and systematic reviews have investigated the effects of exercise on circulating myokine concentrations in the general population (Letukienė et al., 2024), sex‐specific responses to exercise in adults with obesity remain understudied. Accumulating evidence suggests that muscle‐derived signaling pathways are influenced by biological sex hormones, and sex differences in myokine production and action have been reported (Velez et al., 2022). Recent integrative analyses have demonstrated that, while overall myokine expression levels may not markedly differ between sexes, their cross‐tissue signaling and functional interactions are highly sex‐dependent and strongly influenced by sex steroid hormones, particularly estrogen. Importantly, in adults with obesity, who exhibit elevated levels of both muscle and fat mass, disentangling the respective contributions of muscle‐derived myokines and adipose tissue–derived cytokines (adipokines) is challenging, underscoring the need for a controlled study design with matched body composition.

Therefore, this study aimed to compare serum myokine levels in adults with obesity matched for body composition, and to determine whether sex‐specific differences in circulating myokine concentrations are influenced by regular exercise habits. This study assessed fasting circulating myokine concentrations, reflecting basal levels, and specifically focused on skeletal muscle‐derived factors, including MST, FST, irisin, BDNF, FSTL1, and SPARC, to determine whether distinct sex‐specific myokine response profiles exist. Given that body composition and arterial stiffness are associated with myokine regulation and are known to exhibit sex differences, we hypothesized that circulating myokine concentrations would show sex‐specific associations with exercise habits and related physiological factors. However, given the exploratory nature of this study, no specific directional hypotheses were predefined.

## 2 METHODS

### 2.1 Subjects

This study included 226 patients with obesity, defined as a body mass index (BMI) ≥ 30 kg/m^2^, who visited the outpatient clinic of obesity at Kansai Medical University Hospital between January 2016 and March 2021. Specifically, participants were retrospectively identified from patients who attended the obesity clinic for the first time during the study period and were selected using predefined criteria and random sampling procedures. Eighty participants were selected, consisting of 20 males and 20 females with regular exercise habits and 20 males and 20 females without regular exercise habits, matched for age and body composition. Biological sex was determined based on medical records obtained at the initial clinical visit. In addition, regular exercise habits were defined according to the criteria used in the Japanese National Health and Nutrition Survey, in which exercise habits are defined as engaging in exercise for approximately 30 min or more at least twice per week, based on self‐reported exercise behavior (Nakagata & Ono, 2024). This definition does not restrict exercise modality and includes any structured exercise activities regardless of type (e.g., aerobic or resistance exercise). The exclusion criteria were a history of cardiac pacemaker implantation, pregnancy, severe liver dysfunction, renal disease, secondary causes of obesity due to endocrine disorders, and wasting diseases. This study was exploratory, and no a priori sample size calculations were performed.

The study was conducted in accordance with the principles of the Declaration of Helsinki, and all procedures were approved by the Ethics Committee of Kansai Medical University (approval no. 2019092). Written informed consent was obtained from all participants before the study initiation.

### 2.2 Study design

**FIGURE 1:**
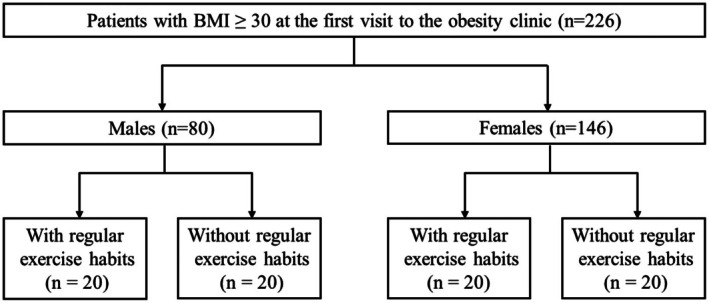
Study protocol. (1) Twenty male and female patients with regular exercise habits were randomly selected. (2) Patients without regular exercise habits were individually matched for age and body composition (body weight, BMI, fat mass, and skeletal muscle mass).

This was a retrospective, sex‐stratified, matched case–control study (Figure 1). Participants were stratified according to biological sex. Among the patients with regular exercise habits, 20 males and 20 females were randomly selected. Subsequently, patients without regular exercise habits were individually matched (1:1) to those with regular exercise habits by selecting individuals within ±3 years of age and with the closest overall similarity across body composition variables, including body weight, BMI, fat mass, and lean body mass.

### 2.3 Measurement

#### 2.3.1 Body composition

Body composition was measured by dual‐energy X‐ray absorptiometry (DXA; DPX‐NT System; GE Healthcare, Buckinghamshire, UK). The measurement parameters included body weight, body fat mass, and lean mass (whole body, upper extremities, trunk, and lower extremities). Percent body fat mass and percent lean body mass were calculated as each value divided by body weight. Skeletal muscle index (SMI) was calculated as appendicular lean body mass (kg) divided by height squared (m^2^). Computed tomography and fat scan analysis software (East Japan Technology Tokyo Laboratory, Tokyo, Japan) were used to measure visceral fat area (VFA) and subcutaneous fat area (SFA) at the umbilical level.

#### 2.3.2 Physical function and arterial stiffness

Physical function was measured based on exercise tolerance and lower limb muscle strength. A symptom‐limited cardiopulmonary exercise (CPX) test was performed using a cycle ergometer (AEROBIKE 75XL; Combi Co., Ltd., Japan). After 4 min of rest on the ergometer, exercise was initiated with a 4‐min warm‐up at 10–20 watts and 50 rpm, followed by the 10–20 watts/min ramp protocol. The ramp rate (10 or 20 W/min) was individually determined to ensure that each participant reached volitional exhaustion within approximately 10 min. CPX was performed under symptom‐limited conditions, and the test was terminated when the participant was unable to maintain a pedaling cadence of at least 50 rpm. Oxygen uptake (VO_2_), carbon dioxide output, and minute ventilation on a breath‐by‐breath basis were measured using an expired gas analyzer (AE‐300 s and 310 s; Minato Medical Science Co., Ltd., Japan). The anaerobic threshold (AT) was determined using the V‐slope method (Beaver et al., 1986). Peak VO_2_ and heart rate were defined as the peak values during incremental exercise.

Lower limb muscle strength was measured twice by using a cycle‐based leg strength ergometer (StrengthErgo; Mitsubishi Electric Engineering Co., Ltd., Tokyo, Japan). Measurements were performed in isokinetic mode at a constant rotation speed of 50 rpm using five consecutive pedaling cycles, and peak torque during bilateral pedaling movements was recorded. The higher value was normalized to body weight (N·m/kg) and used for analysis.

Arterial stiffness was assessed by measuring brachial‐ankle pulse wave velocity (baPWV), and values were expressed in meters per second (m/s). After 10 min of rest in the supine position, baPWV was measured using a pulse pressure analyzer (BP‐203RPE; Omron Colin Co. Ltd., Japan). Measurements were performed twice at 2‐min intervals, and the mean value from the right and left arms was considered the final baPWV.

#### 2.3.3 Physical activity

Physical activity was self‐reported using the shortened version of the International Physical Activity Questionnaire (IPAQ) (Craig et al., 2003). The questionnaire consisted of seven questions that assessed physical activity in the past week. Metabolic equivalent (MET) values for physical activity were classified as light (3.3 MET), moderate (4.0 MET), and high (8.0 MET). Daily physical activity and walking time were calculated by aggregating activities lasting ≥10 min based on reported frequency and intensity. The sitting time was defined as the average daily sedentary time between waking and sleeping.

#### 2.3.4 Blood sampling

Blood samples were collected to determine serum levels of high‐density lipoprotein (HDL) cholesterol, low‐density lipoprotein (LDL) cholesterol, triglycerides, fasting blood glucose, glycosylated hemoglobin (HbA1c), C‐reactive protein (CRP), and immunoreactive insulin (IRI). Homeostasis model assessment of insulin resistance (HOMA‐IR) was performed using the following formula: HOMA‐IR = (IRI × fasting plasma glucose)/405. Blood samples were stored at −80°C, and myokine levels were measured according to the manufacturer's instructions.

Serum MST, FST, and BDNF levels were measured using human Quantikine ELISA Kits (R&D Systems, Minneapolis, MN, USA; catalog numbers DGDF80, DFN00, and DBD00). Serum irisin levels were measured using a human EIA kit (Phoenix Pharmaceuticals Inc., Burlingame, CA, USA; catalog no. EK‐067‐29). Serum FSTL1 and SPARC levels were measured using human FSTL1 and SPARC ELISA Kits (Catalog No. ab302766 and ab220654).

The intra‐ and inter‐assay coefficients of variation were 1.8%–5.4% and 3.6%–6.0% for MST, 2.0%–2.7% and 7.9%–9.2% for FST, 5.0%–6.2% and 8.1%–11.3% for BDNF, <10% and <15% for irisin, 13.0% and 12.8% for FSTL1, and 2.3% and 4.5% for SPARC, respectively.

### 2.4 Statistical analysis

Data are expressed as means ± standard deviation or median (interquartile range), depending on the data distribution, and categorical variables are presented as numbers and percentages. The Shapiro–Wilk test was used to assess normality. Comparisons between participants with and without regular exercise habits were performed using the chi‐square test for categorical variables, and effect sizes were calculated using Cramér's *V*. A two‐way analysis of variance (ANOVA) was used to evaluate the main effects of sex and exercise habits and their interaction. When a significant interaction was observed, post hoc comparisons were performed using the Bonferroni correction. Variables that were normally distributed were analyzed without transformation, whereas variables with non‐normal distributions were log‐transformed prior to analysis to satisfy the assumptions of ANOVA. Effect sizes (*η* ^*2*^) were calculated for each main effect and interaction.

These analyses were exploratory and conducted based on the results of the initial group comparisons. Subsequent analyses focused on the myokines that demonstrated significant sex differences and differences according to exercise habits. Multiple regression analyses were performed to identify the independent determinants of serum myokine levels. The independent variables that showed significant interactions in the two‐way ANOVA, along with clinically relevant covariates (age, percent lean body mass, exercise tolerance, and insulin resistance), were entered into multiple linear regression models using a stepwise selection method, given the exploratory nature of the study, limited sample size, and relatively large number of candidate variables. Collinearity was assessed using the variance inflation factor (VIF), and variables with VIF >5 were excluded from the model.

All statistical analyses were performed using SPSS (version 26.0, IBM Corp., Armonk, NY, USA), and a two‐sided *p* < 0.05 was considered statistically significant.

## 3 RESULTS

### 3.1 Patient characteristics by exercise habit status and sex

The baseline patient characteristics stratified by exercise habit status and sex are presented in Table 1. A significant main effect of exercise habits was observed for hypertension (*p* = 0.007, *V* = 0.30), with a lower prevalence in the exercise group. Although several continuous variables showed expected sex differences, no significant sex × exercise interactions were observed.

**TABLE 1:** Baseline characteristics of participants.

	Males		Females		Sex	Exercise	Interaction
	Exercise (+)	Exercise (−)	Exercise (+)	Exercise (−)	*p* Value (*η* ^*2*^ or *V*)	*p* Value (*η* ^*2*^ or *V*)	*p* Value (*η* ^*2*^)
Age (years)	41.9 ± 12.5	43.0 ± 12.2	47.8 ± 16.6	49.3 ± 10.7	0.041 (0.05)	0.654 (0.00)	0.939 (0.00)
Height (cm)	173.1 ± 6.0	171.3 ± 4.4	159.6 ± 5.6	158.9 ± 6.7	<0.001 (0.57)	0.313 (0.01)	0.673 (0.00)
Weight (kg)	110.1 ± 14.3	112.0 ± 18.3	87.5 ± 13.8	88.9 ± 13.0	<0.001 (0.36)	0.656 (0.00)	0.775 (0.00)
BMI (kg/m^2^)	36.8 ± 4.8	38.5 ± 7.2	34.7 ± 4.9	35.3 ± 5.1	0.039 (0.06)	0.345 (0.01)	0.649 (0.00)
Hypertension, *n* (%)	7 (35.0)	12 (60.0)	5 (25.0)	12 (60.0)	0.653 (0.05)	0.007 (0.30)	N/A
Dyslipidemia, *n* (%)	6 (30.0)	5 (25.0)	4 (20.0)	7 (35.0)	1.000 (0.00)	0.617 (0.06)	N/A
Diabetes, *n* (%)	3 (15.0)	8 (40.0)	7 (35.0)	10 (50.0)	0.160 (0.16)	0.061 (0.21)	N/A
Antihypertensive medication, *n* (%)	6 (30.0)	9 (45.0)	4 (20.0)	7 (35.0)	0.340 (0.11)	0.152 (0.16)	N/A
Lipid‐lowering medication, *n* (%)	5 (25.0)	3 (15.0)	4 (20.0)	5 (25.0)	0.785 (0.03)	0.785 (0.03)	N/A
Insulin, *n* (%)	0 (0.0)	0 (0.0)	0 (0.0)	1 (5.0)	0.314 (0.11)	0.314 (0.11)	N/A
Oral hypoglycemic agents, *n* (%)	3 (15.0)	8 (40.0)	4 (20.0)	5 (25.0)	0.606 (0.06)	0.121 (0.17)	N/A
Smoking, *n* (%)	4 (20.0)	2 (10.0)	4 (20.0)	1 (5.3)	0.780 (0.03)	0.114 (0.18)	N/A
Alcohol consumption, *n* (%)	9 (45.0)	6 (30.0)	8 (42.1)	4 (21.1)	0.583 (0.06)	0.096 (0.19)	N/A

*Note*: Data are expressed as means ± standard deviation or *n* (%). Continuous variables were analyzed using two‐way analysis of variance (ANOVA) to evaluate the main effects of sex and exercise habits and their interaction. Effect sizes are presented as partial *η* ^*2*^. Categorical variables were analyzed using chi‐square tests. *p* Values are presented for comparisons according to sex and exercise habits. Effect sizes are presented as Cramér's *V*. Sex × exercise interaction was not assessed for categorical variables.

Abbreviations: BMI, body mass index; N/A, not applicable.

The body composition, physical function, physical activity levels, and blood biochemical parameters are shown in Table 2. A significant interaction between sex and exercise habits was observed for baPWV (*p* = 0.031, *η* ^*2*^ = 0.06), daily physical activity (*p* = 0.035, *η* ^*2*^ = 0.06), and LDL‐cholesterol (*p* = 0.049, *η* ^*2*^ = 0.05). Post hoc comparisons with Bonferroni correction did not reveal significant differences between groups for LDL‐cholesterol. A main effect of sex was observed for percent body fat mass (*p* < 0.001, *η* ^*2*^ = 0.41), lean body mass (*p* < 0.001, *η* ^*2*^ = 0.72), percent lean body mass (*p* < 0.001, *η* ^*2*^ = 0.39), SMI (*p* < 0.001, *η* ^*2*^ = 0.48), Peak VO_2_ (*p* = 0.016, *η* ^*2*^ = 0.07), isokinetic leg strength (*p* < 0.001, *η* ^*2*^ = 0.30), daily physical activity (*p* < 0.001, *η* ^*2*^ = 0.15), HDL‐cholesterol (*p* < 0.001, *η* ^*2*^ = 0.18), insulin (*p* < 0.001, *η* ^*2*^ = 0.14), HOMA‐IR (*p* < 0.001, *η* ^*2*^ = 0.15), and CRP (*p* = 0.036, *η* ^*2*^ = 0.06). A main effect of exercise habits was observed for AT (*p* < 0.001, *η* ^*2*^ = 0.24), Peak VO_2_ (*p* < 0.001, η^2^ = 0.24), sedentary time (*p* = 0.046, *η* ^*2*^ = 0.05), fasting glucose (*p* = 0.016, η^2^ = 0.07), insulin (*p* = 0.010, *η* ^*2*^ = 0.09), and HOMA‐IR (*p* = 0.004, *η* ^*2*^ = 0.11).

**TABLE 2:** Comparison of body composition, physical function, physical activity level, and laboratory blood measurements.

	Males		Females		Sex	Exercise	Interaction
	Exercise (+)	Exercise (−)	Exercise (+)	Exercise (−)	*p*‐value (*η* ^*2*^)	*p*‐value (*η* ^*2*^)	*p*‐value (*η* ^*2*^)
Body composition							
Body fat mass (kg)	43.0 ± 10.7	43.6 ± 13.1	40.8 ± 8.9	43.3 ± 10.9	0.442 (0.01)	0.681 (0.00)	0.850 (0.00)
Percent body fat mass (%)	38.7 ± 5.9	38.2 ± 5.8	46.3 ± 3.4	47.5 ± 5.3	<0.001 (0.41)	0.895 (0.00)	0.531 (0.01)
Lean body mass (kg)	66.1 ± 7.6	67.5 ± 6.6	46.0 ± 5.7	46.6 ± 6.7	<0.001 (0.72)	0.572 (0.00)	0.705 (0.00)
Percent lean body mass (%)	60.4 ± 6.1	61.0 ± 5.8	52.8 ± 3.6	52.3 ± 5.1	<0.001 (0.39)	0.970 (0.00)	0.617 (0.00)
SMI (kg/m^2^)	9.2 ± 1.2	9.1 ± 1.2	7.2 ± 1.1	7.2 ± 0.9	<0.001 (0.48)	0.649 (0.00)	0.961 (0.00)
VFA (cm^2^)^a^	223.5 (139.7–307.3)	213.3 (164.5–245.7)	153.2 (128.2–177.1)	213.8 (128.7–270.2)	0.023 (0.07)	0.136 (0.03)	0.128 (0.03)
SFA (cm^2^)^a^	418.6 (285.5–564.4)	393.0 (285.3–539.5)	397.2 (368.8–525.8)	402.4 (296.6–540.1)	0.984 (0.00)	0.630 (0.00)	0.590 (0.00)
Physical function							
AT (mL/kg/min)	12.6 ± 2.5	10.5 ± 1.6	12.2 ± 2.3	10.1 ± 1.5	0.302 (0.01)	<0.001 (0.24)	0.929 (0.00)
Peak VO_2_ (mL/kg/min)	22.0 ± 4.7	18.2 ± 3.7	20.2 ± 3.8	15.9 ± 2.9	0.016 (0.07)	<0.001 (0.24)	0.648 (0.00)
Isokinetic leg strength (N·m/kg)	1.7 ± 0.3	1.7 ± 0.3	1.4 ± 0.2	1.3 ± 0.2	<0.001 (0.30)	0.644 (0.00)	0.722 (0.00)
baPWV (m/s)^a^	13.31 (12.24–14.13)	12.61 (11.60–14.44)	11.97 (11.10–14.66)	14.02 (12.57–16.31)	0.310 (0.01)	0.093 (0.04)	0.031 (0.06)
Physical activity level							
Daily physical activity (kcal/day)^a^	290.6 (205.2–1160.5)	0.0 (0.0–38.2)	312.9 (185.9–532.8)	0.0 (0.0–0.0)	<0.001 (0.15)	<0.001 (0.85)	0.035 (0.06)
Sedentary time (min/day)^a^	345 (240–600)	660 (345–855)	480 (255–600)	600 (300–840)	0.908 (0.00)	0.046 (0.05)	0.484 (0.01)
Laboratory blood measurment							
HDL‐Cholesterol (mg/dL)	41.7 ± 8.4	41.8 ± 9.0	51.5 ± 9.6	50.0 ± 12.8	<0.001 (0.18)	0.765 (0.00)	0.748 (0.00)
LDL‐Cholesterol (mg/dL)	125.5 ± 40.3	109.2 ± 26.2	105.4 ± 28.1	118.9 ± 35.6	0.482 (0.01)	0.843 (0.00)	0.049 (0.05)
Triglycerides (mg/dL)	132.5 ± 56.6	140.1 ± 59.7	92.4 ± 31.8	132.1 ± 92.8	0.134 (0.03)	0.069 (0.04)	0.193 (0.02)
Fasting glucose (mg/dL)	99.0 ± 12.0	109.4 ± 19.0	95.8 ± 15.1	101.3 ± 13.3	0.114 (0.03)	0.016 (0.07)	0.530 (0.01)
HbA1c (%)	5.7 ± 0.5	6.0 ± 0.7	5.9 ± 0.6	6.1 ± 0.6	0.174 (0.02)	0.052 (0.05)	0.613 (0.00)
Insulin (μU/mL)^a^	16.1 (14.0–19.4)	18.0 (12.0–26.0)	8.8 (6.5–10.9)	13.0 (10.7–18.5)	<0.001 (0.14)	0.010 (0.09)	0.216 (0.02)
HOMA‐IR^a^	3.8 (3.1–4.5)	4.7 (3.1–7.6)	1.9 (1.5–2.6)	3.5 (2.6–4.6)	<0.001 (0.15)	0.004 (0.11)	0.144 (0.03)
CRP (mg/dL)^a^	0.02 (0.08–0.25)	0.17 (0.05–0.41)	0.19 (0.09–0.47)	0.23 (0.10–0.52)	0.036 (0.06)	0.495 (0.01)	0.954 (0.00)

*Note*: Data are expressed as means ± standard deviation or median (interquartile range).

Abbreviations: AT, anerobic threshold; baPWV, brachial‐ankle pulse wave velocity; CRP, C‐reactive protein; HbA1c, glycated hemoglobin; HDL, high density lipoprotein; HOMA‐IR, homeostasis model assessment of insulin resistance; LDL, low density lipoprotein; SFA, subcutaneous fat area; SMI, skeletal muscle mass index; VFA, visceral fat area; VO_2_, oxygen consumption.

^a^ Variables with non‐normal distributions were log‐transformed prior to analysis. *p* Values and effect sizes (*η* ^*2*^) were obtained from a two‐way ANOVA.

### 3.2 Serum myokine concentrations by exercise habit status in males and females

**FIGURE 2:**
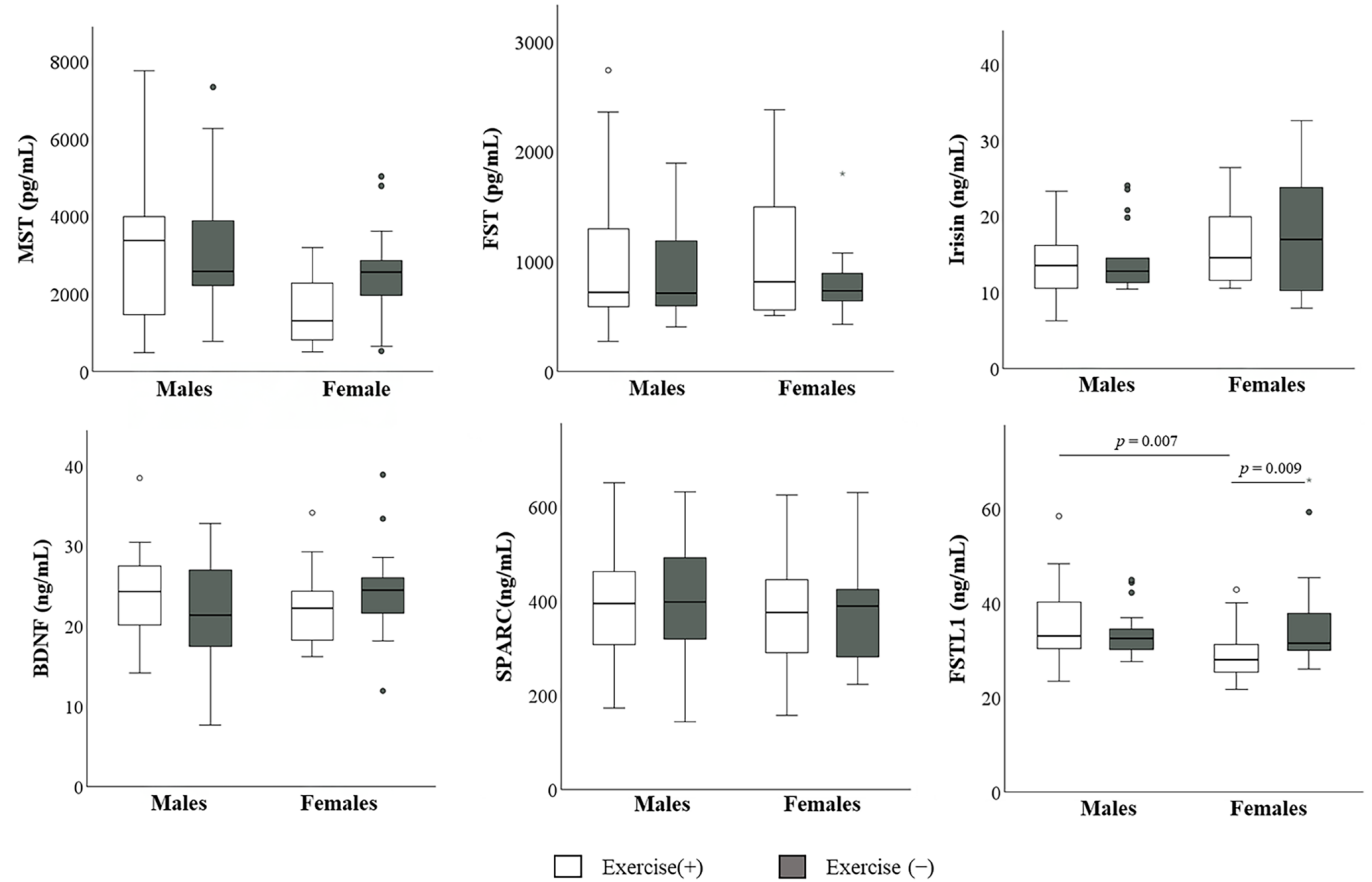
Comparison of serum myokine levels by exercise habit status in males and females. Box‐and‐whisker plots show serum myokine levels stratified by sex and exercise habits. Boxes represent the interquartile range, the horizontal line within each box indicates the median, and whiskers indicate the range. A significant interaction between sex and exercise habits was observed for FSTL1. Post hoc comparisons with Bonferroni correction indicated that FSTL1 levels were significantly lower in females with exercise habits than in females without exercise habits and males with exercise habits. No significant interaction was observed for MST; however, significant main effects of sex and exercise habits were identified.

Serum myokine concentrations stratified by sex and exercise habits are shown in Figure 2 and Table S1. A significant interaction between sex and exercise habits was observed for FSTL1 (*p* = 0.023, *η* ^*2*^ = 0.07). Post hoc comparisons with Bonferroni correction revealed that serum FSTL1 levels in females with exercise habits were significantly lower than those in females without exercise habits (median: 28.1 vs. 31.6 ng/mL, *p* = 0.009, *η* ^*2*^ = 0.09) and were also significantly lower than those in males with exercise habits (median: 28.1 vs. 33.0 ng/mL, *p* = 0.007, *η* ^*2*^ = 0.09). In contrast, no significant interaction was observed for MST. However, significant main effects of sex (*p* = 0.004, *η* ^*2*^ = 0.10) and exercise habits (*p* = 0.030, *η* ^*2*^ = 0.06) were identified for MST.

### 3.3 Independent factors associated with MST and FSTL1 in males and females

To identify independent determinants of MST and FSTL1, multivariate linear regression analyses were performed separately in males and females. Independent variables were selected based on patient characteristics analyses, including factors showing significant interaction effects in the two‐way ANOVA, along with clinically relevant covariates (Table 3). In males, Peak VO_2_ (*β* = −0.461, *p* = 0.003, *R* ^*2*^ = 0.19) was identified as an independent determinant of MST, whereas LDL‐cholesterol (β = 0.408, *p* = 0.009, *R* ^*2*^ = 0.14) was independently associated with FSTL1. In females, daily physical activity was identified as an independent determinant of both MST (β = −0.400, *p* = 0.011, *R* ^*2*^ = 0.14) and FSTL1 (β = −0.417, *p* = 0.007, *R* ^*2*^ = 0.15).

**TABLE 3:** Stepwise multiple regression analyses with MST and FSTL‐1 in males and females.

	Males (*n* = 40)			Females (*n* = 40)		
	β	*p* Value	VIF	β	*p* Value	VIF
(A) Dependent variable: log_MST						
Daily physical activity^a^	−0.040	0.589	1.090	**−0.400**	**0.011**	**1.000**
baPWV^a^	0.083	0.786	1.010	−0.140	0.397	1.190
LDL‐Cholesterol	0.167	0.253	1.000	0.004	0.981	1.053
Age	0.067	0.937	1.026	0.078	0.610	1.019
Percent lean body mass	0.079	0.661	1.080	0.045	0.766	1.006
Peak VO_2_	**−0.461**	**0.003**	**1.000**	−0.102	0.583	1.515
HOMA‐IR^a^	−0.012	0.590	1.007	−0.095	0.587	1.346
(B) Dependent variable: log_FSTL1						
Daily physical activity^a^	0.071	0.648	1.052	**−0.417**	**0.007**	**1.000**
baPWV^a^	0.194	0.194	1.001	−0.052	0.750	1.190
LDL‐Cholesterol	**0.408**	**0.009**	**1.000**	0.117	0.448	1.053
Age	0.191	0.206	1.020	0.056	0.713	1.019
Percent lean body mass	−0.062	0.688	1.065	0.067	0.656	1.006
Peak VO_2_	−0.019	0.201	1.000	−0.186	0.312	1.515
HOMA‐IR^a^	−0.035	0.819	1.013	0.261	0.129	1.346

*Note*: Values are presented as standardized regression coefficients (*β*). Independent variables included factors showing significant interactions in the two‐way ANOVA, along with adjustment variables (age, percent fat‐free mass, exercise tolerance, and insulin resistance). Bold values indicate statistically significant differences (*p* < 0.05).

Abbreviations: FSTL1, follistatin‐like 1; HOMA‐IR, homeostasis model assessment of insulin resistance; LDL, low density lipoprotein; MST, myostatin; PWV, pulse wave velocity; VO_2_, oxygen consumption.

^a^ Log‐transformed before inputting.

## 4 DISCUSSION

### 4.1 Main findings

This study examined the association between exercise habits and serum myokine concentrations in adults with obesity matched for body composition, with analyses stratified by sex. The principal findings of this study were that a significant interaction between sex and exercise habits was observed for FSTL1, whereas MST showed significant main effects of both sex and exercise habits. Serum FSTL1 levels were lower in females with exercise habits than in females without exercise habits and were also lower than those in males with exercise habits, whereas MST showed only main effects without a significant interaction. Furthermore, multivariate analysis revealed that daily physical activity was an independent determinant of both MST and FSTL1 in females, whereas Peak VO_2_ and LDL‐cholesterol were identified as independent determinants in males.

### 4.2 Sex differences and exercise habits in FSTL1 and MST levels

Previous studies have demonstrated that myokine secretion is influenced by changes in body weight and composition and that secretion dynamics vary according to the rate of weight loss and type of intervention employed (Kurose et al., 2024). In the present study, a significant interaction between sex and exercise habits was observed for FSTL1, indicating a sex‐specific response. Animal studies have shown that aerobic exercise increases FSTL1 expression, which is positively correlated with arterial nitrite/nitrate (NOx) levels (Inoue et al., 2022). Conversely, human studies have reported higher circulating FSTL1 levels in patients with coronary artery disease (CAD) compared to those without CAD, with progressive increases in the number of coronary vessels exhibiting >50% stenosis (Yamazaki et al., 2021). Furthermore, weight loss achieved through physical activity or dietary intervention has been reported to reduce FSTL1 levels in parallel with improvements in glucose metabolism (Tok et al., 2021). Collectively, these findings suggest that FSTL1 may exhibit dual characteristics, being upregulated protectively in response to exercise stimulation, while also increasing compensatorily in the presence of metabolic dysfunction and vascular pathology. In this study, we found that females with obesity who reported regular exercise habits exhibited lower FSTL1 levels, despite comparable body composition. Multivariate analysis further identified daily physical activity as an independent determinant of FSTL1 levels in females, whereas LDL‐cholesterol was independently associated with FSTL1 in males. These findings suggest that habitual physical activity may contribute to the regulation of FSTL1 levels in females, while lipid‐related factors may play a more prominent role in males. From a mechanistic perspective, FSTL1 has been reported to be involved in vascular protection and inflammation (Peters et al., 2019). Increased FSTL1 levels under pathological conditions such as atherosclerosis may reflect a compensatory response to vascular stress (Ponce‐Ruíz et al., 2024). Therefore, the lower FSTL1 levels observed in females with regular exercise habits may indicate reduced compensatory activation due to improved physiological conditions associated with habitual physical activity. In contrast, no significant differences in FSTL1 levels were observed among males according to exercise habits, suggesting that the regulatory mechanisms of FSTL1 may differ between sexes, potentially due to differences in hormonal environment, fat distribution, and muscle–adipose tissue crosstalk.

In parallel with the sex‐specific regulation observed for FSTL1, MST also showed significant main effects of sex and exercise habits in the present study. Notably, a study in which participants maintained their body composition through dietary adjustments while continuing aerobic exercise reported a significant reduction in serum MST levels (Hittel et al., 2010), which supports the findings observed in females with obesity in the present study. These results suggest that habitual daily physical activity may contribute to the regulation of MST, even when body composition is comparable. In the present study, sex‐specific determinants of MST were identified. Multivariate analysis revealed that Peak VO_2_ was an independent determinant of MST levels in males, whereas daily physical activity was the sole independent determinant in females, with both showing inverse associations. In males with obesity, the inverse association between Peak VO_2_ and MST suggests that MST levels may be more closely linked to exercise tolerance and aerobic capacity, reflecting adaptations to higher‐intensity or more physiologically demanding physical activity. This finding implies that improving cardiorespiratory fitness, rather than merely increasing activity volume, may be important for the regulation of MST in males. In contrast, in females with obesity, the inverse association between daily physical activity and MST indicates that overall activity volume, regardless of exercise intensity, may play a more prominent role in MST regulation. These findings suggest that MST in females may be more sensitive to cumulative muscle contraction stimuli derived from habitual daily activity than to aerobic capacity. Although previous studies have reported associations among MST, appendicular skeletal muscle mass, and insulin levels (Kurose et al., 2021; Zhang et al., 2025), these variables were not identified as independent predictors in the present analysis, highlighting the predominant role of physical activity–related factors. Although sex hormones and menopausal status were not evaluated in this study, estrogen has been reported to modulate MST signaling (Pellegrino et al., 2022), suggesting that the hormonal milieu may partly contribute to these sex‐specific responses. Collectively, these findings indicate that the regulatory mechanisms of MST differ between sexes, with exercise capacity–related factors being more influential in males, whereas habitual physical activity may play a more dominant role in females.

### 4.3 Strengths and limitations

A major strength of this study is the matching of body composition between groups according to exercise habits, which enabled the evaluation of myokine levels independent of physique. This approach revealed sex‐specific associations of myokines with physiological and lifestyle‐related factors, showing that daily physical activity was negatively associated with both MST and FSTL1 levels in females with obesity, whereas Peak VO_2_ was negatively associated with MST and lipid‐related factors such as LDL‐cholesterol was positively associated with FSTL1 in males. These findings highlight distinct regulatory pathways of myokines between sexes, suggesting that habitual physical activity may play a central role in females, whereas cardiorespiratory fitness and lipid‐related factors may be more influential in males. This may offer potential applications for exercise guidance in clinical settings.

This study had several limitations. First, as this was a retrospective cross‐sectional study, causality could not be established. Although participants were matched for age and body composition, residual sex‐related differences may remain due to inherent biological variation. Therefore, the observed sex differences in myokine levels should be interpreted with consideration of these factors. Second, exercise habits were assessed using self‐reported questionnaires that lacked detailed information on exercise type, intensity, and frequency. Third, the sample size was relatively small, and multivariate analyses were exploratory in nature and potentially affected by model instability due to stepwise selection. Fourth, sex hormone concentrations, menopausal status, hormonal contraceptive use, and hormone replacement therapy were not available. As sex hormones may influence circulating myokine levels, these unmeasured factors could have affected the observed sex‐specific associations. Future prospective studies or large‐scale cohort studies are required to evaluate sex hormone levels and exercise characteristics in greater detail. Finally, as this study used serum samples, the tissue source of myokine secretion could not be identified, and it cannot be ruled out that the findings may not necessarily reflect gene expression at the tissue level. Despite these limitations, the matched study design strengthened the validity of the observed sex‐specific associations.

## 5 CONCLUSION

In adults with obesity matched for body composition, a comparison of serum myokine levels according to sex and exercise habits demonstrated that serum FSTL1 levels were significantly lower in exercise‐habituated females than in their non‐exercise‐habituated counterparts, indicating a clear sex‐specific difference. In contrast, MST showed significant main effects of sex and exercise habits. Furthermore, daily physical activity was identified as an independent determinant of MST and FSTL1 in females, whereas Peak VO_2_ and LDL‐cholesterol were independently associated with MST and FSTL1, respectively, in males. These findings suggest that MST may be associated with exercise‐related muscular activity or physiological adaptation, whereas FSTL1 may reflect lipid‐related vascular and metabolic status in a sex‐dependent manner. Collectively, these results highlight distinct regulatory mechanisms of myokines between sexes and underscore the importance of sex‐specific approaches in exercise interventions for individuals with obesity.

## AUTHOR CONTRIBUTIONS

**Satoshi Kurose:** Conceptualization; data curation; funding acquisition; methodology. **Katsuko Onishi:** Data curation; methodology. **Takumi Miyauchi:** Investigation; validation. **Kazuhisa Takahashi:** Data curation; methodology; supervision. **Yutaka Kimura:** Investigation; methodology; supervision.

## FUNDING INFORMATION

This study was supported by the Japan Society for the Promotion of Science (JSPS) through a Grant‐in‐Aid for Scientific Research (KAKENHI) (Grant Number JP25K14973).

## CONFLICT OF INTEREST STATEMENT

The authors declare that they have no competing interests.

## ETHICS STATEMENT

The study was conducted in accordance with the principles of the Declaration of Helsinki, and all procedures were approved by the Ethics Committee of Kansai Medical University (approval no. 2019092). Written informed consent was obtained from all participants before study initiation.

## Supporting information


Table S1.

## Data Availability

Data are available from the corresponding author upon request.
