# Supplementary material for: Sex‐specific associations between regular exercise habits and serum myokine levels in adults with obesity
Source: Physiol Rep. 2026 May 27;14(10):e70930. doi: 10.14814/phy2.70930 (PMC13239317; doi:10.14814/phy2.70930)
Supplement: Supplementary file 1 — Table S1. [file PHY2-14-e70930-s001.docx]

**Supplementary Table 1. Two-way ANOVA results for log-transformed serum myokine levels**

These variables with non-normal distributions were log-transformed prior to analysis. *p*-values and effect sizes (*η²*) were obtained from a two-way ANOVA.

MST, myostatin; FST, follistatin, BDNF, brain-derived neurotrophic factor; SPARC, secreted protein acidic and rich in cysteine; FSTL1, follistatin-related protein 1.
